# Supplementary material for: Dectin-1 ligands produce distinct training phenotypes in human monocytes through differential activation of signaling networks
Source: Sci Rep. 2024 Jan 17;14:1454. doi: 10.1038/s41598-024-51620-8 (PMC10791629; doi:10.1038/s41598-024-51620-8)
Supplement: Supplementary file 1 — Supplementary Figures. [file 41598_2024_51620_MOESM1_ESM.pdf]

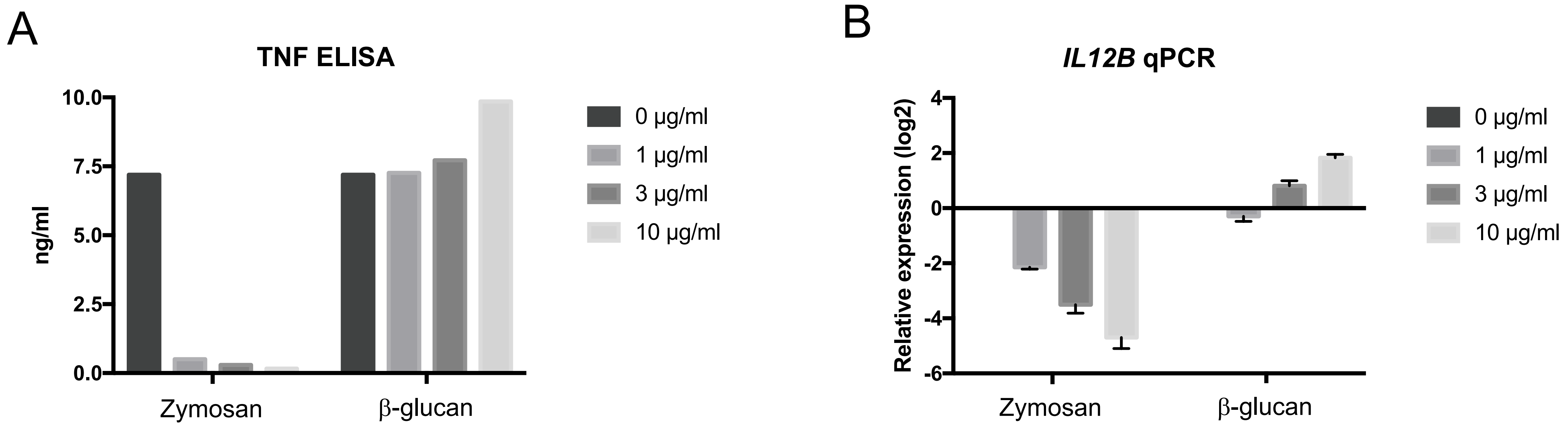

Figure S1: Differential training effects are not a function of dose. (A) TNF ELISA from supernatants of macrophages stimulated with LPS for eight hours, after training with the indicated doses of zymosan or β-glucan from ETSU. **(B)** qPCR for *IL12B* in macrophages, same conditions as (A).

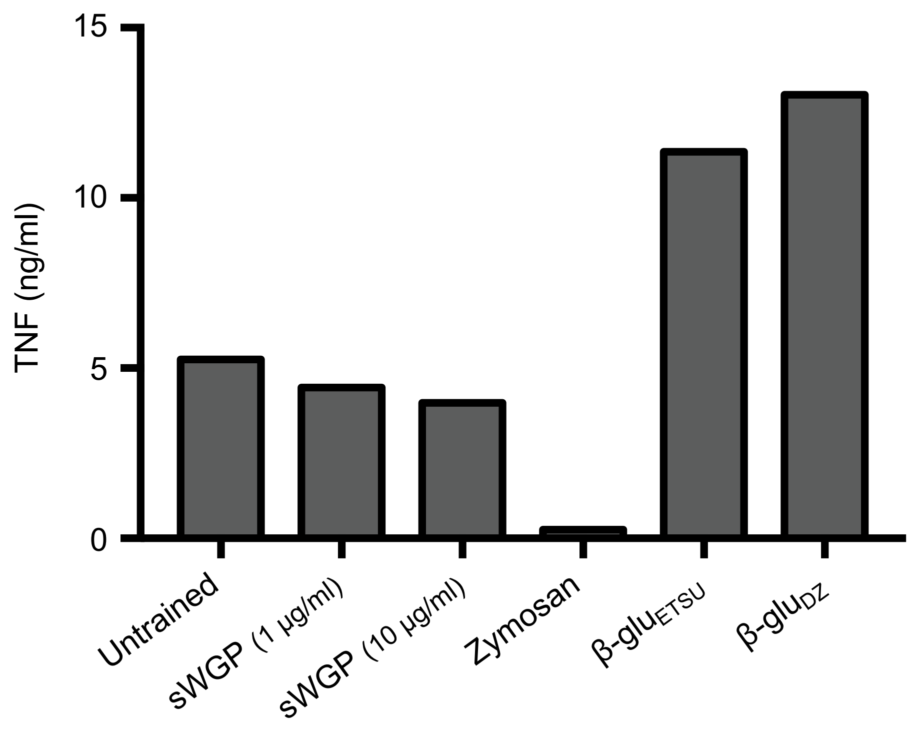

Figure S2: Soluble β-glucans do not induce immune training. TNF ELISA from supernatants of macrophages stimulated with LPS for eight hours, after training with the indicated ligands; sWGP = soluble whole glucan particle, β-glu$_{ETSU}$ = β-glucan from East Tennessee State University, β-glu$_{DZ}$ = depleted zymosan

A

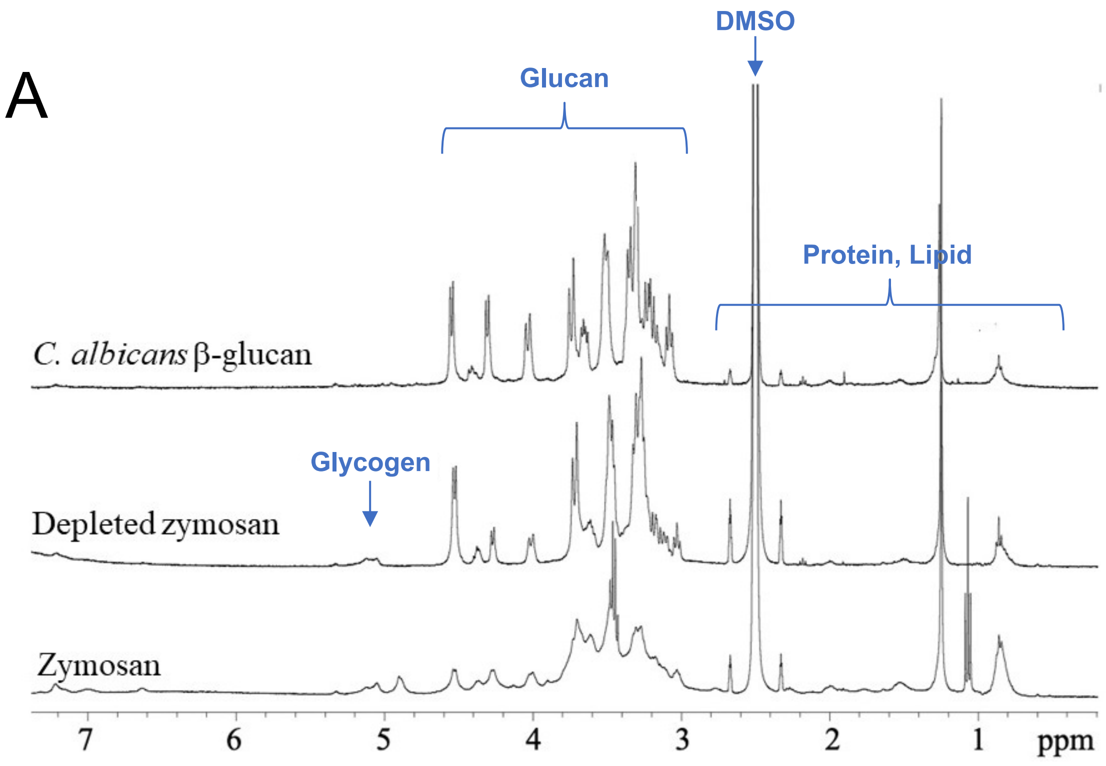

B

	% carbohydrate	% glycogen	% glucan	1-6 sidechain length	branching frequency
**Zymosan**	ND	29%	ND	ND	ND
**Depleted zymosan**	85%	11%	80%	4.2 repeats	12.4 repeats
**ETSU β glucan**	~100%	0%	~100%	12.8 repeats	14.6 repeats

Figure S3: NMR analysis of Dectin-1 ligands. (A) NMR traces of each ligand. **(B)** Table of features of the carbohydrate component derived from NMR analysis; ND = unable to determine.

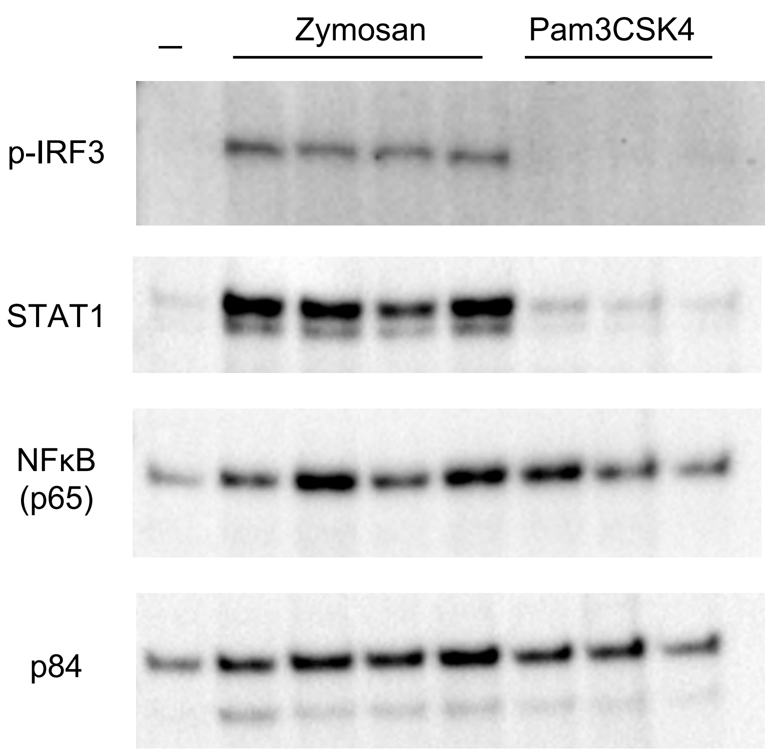

Figure S4: Pam3CSK4 does not activate IRF3-STAT1 axis. Immunoblot of nuclear extracts from Day 0 monocytes stimulated with Zymosan (1 µg/ml) or Pam3CSK4 (100 ng/ml) for two hours; four replicates of each condition.
