# Supplementary material for: Dectin-1 ligands produce distinct training phenotypes in human monocytes through differential activation of signaling networks
Source: Sci Rep. 2024 Jan 17;14:1454. doi: 10.1038/s41598-024-51620-8 (PMC10791629; doi:10.1038/s41598-024-51620-8)
Supplement: Supplementary file 2 — Supplementary Information. [file 41598_2024_51620_MOESM2_ESM.docx]

**Table S1:** Counts table for RNA-seq data in Figure 2, expressed in reads per kilobase of transcript per million reads mapped (RPKM)
